# Progress towards improving mouse models for preclinical testing of treatments for Alzheimer’s disease

**DOI:** 10.1007/s00335-026-10290-9

**Published:** 2026-11-03

**Authors:** Michael Sasner, Claudia Rangel-Barajas, Adrian L. Oblak

**Affiliations:** 1 The Jackson Laboratory, 600 Main Street, Bar Harbor, ME 04609 USA; 2 Department of Medical and Molecular Genetics, Indiana University School of Medicine, Indianapolis, IN 46202 USA; 3 Stark Neurosciences Research Institute, 320 W 15th Street, Indianapolis, IN 46202 USA; 4 Department of Radiology and Imaging Sciences, Indiana University School of Medicine, Indianapolis, IN 46202 USA

## Abstract

Alzheimer’s disease (AD) research has accomplished significant strides in recent years, particularly with the first regulatory approvals of disease-modifying therapies and a comprehensive revision of disease diagnostic criteria. Both developments place new demands on preclinical mouse models used to test potential treatments. This review summarizes recent progress in optimizing mouse models for Alzheimer’s research, using the NIA-funded Model Organism Development and Evaluation for Late-onset Alzheimer’s Disease (MODEL-AD) program as a case study, with specific focus on their relevance for preclinical therapeutic testing, their genetic and molecular fidelity to the human disease, and the contribution of the MODEL-AD program. Key advancements discussed include the shift from transgenic overexpression models to knock-in and genome-replacement strategies; the development of late-onset Alzheimer’s models (LOAD), LOAD1, LOAD2, LOAD3 and related LOAD-platform mice expressing humanized APOE, APP, and MAPT; the integration of environmental risk factors such as high-fat diet and toxic metal exposure into gene x environment interaction models; detection of prodromal stages of disease using multi-omics signatures; and the use of clinically relevant biomarkers, including plasma NfL, GFAP, and p-tau217, as cross-species translational endpoints. We discuss the importance of diverse genetic backgrounds, the contribution of the AD Knowledge Portal as an open-science data-sharing resource, and the principles of rigorous preclinical study design required to maximize translational success. We also discuss the limitations of these models and the evidence still needed to establish their predictive validity for therapeutics. These advances do not yet amount to validated models of late-onset AD, but they give the field better tools and frameworks for selecting and validating mouse models for specific human mechanisms and therapeutic questions.

## Key points

- Animal models based on the genetics of late-onset AD (LOAD), rather than familial AD (fAD), are needed to model mechanisms relevant to the majority of AD patients, who do not carry autosomal dominant mutations; fAD-based models remain valuable for amyloid, CAA, and ARIA biology.
- Knock-in and genome-replacement mouse models expressing humanized variants of APOE, APP, and MAPT at physiological levels avoid the non-physiological expression levels and insertional mutagenesis of transgenic overexpression lines, making them better-controlled platforms for many questions.
- The MODEL-AD program has created a series of progressively refined LOAD platform mice, LOAD1 (APOE4/Trem2*R47H), LOAD2 (APOE4/Trem2*R47H/hAβ), and LOAD3 (APOE4/hAβ/hMAPT), progressively adding major LOAD risk alleles to a common platform.
- Improved genetic (construct) validity does not by itself establish predictive validity: no knock-in LOAD model has yet been benchmarked against clinical trial outcomes, and doing so requires testing drugs with known clinical results.
- Environmental risk factors, including high-fat diet, vascular risk, and toxic metal exposures, can be combined with genetic risk factors and aging to produce clinically relevant disease phenotypes suited for testing therapeutics independent of amyloid and tau pathology.
- Clinically aligned phenotyping modalities such as plasma NfL, GFAP, and p-tau217, in vivo PET/MRI, and touchscreen cognitive assessment, aligned with the 2024 revised Alzheimer’s Association criteria, should be incorporated into mouse model evaluation pipelines to maximize translational utility.
- Genetic diversity across inbred backgrounds, wild-derived strains, and outbred stocks substantially influences the expression of AD phenotypes, including neuroinflammation and cerebral amyloid angiopathy; this diversity must be harnessed rather than minimized.
- Each therapeutic compound should be matched to the right target, in the right model, at the right age, using the right assay, with study designs that mirror clinical trial standards including pre-specified endpoints, randomization, blinding, and inclusion of equal numbers of both sexes.

## Introduction

Dementia represents the leading cause of disability in older adults globally. Alzheimer’s disease (AD) is the most prevalent form, accounting for 60–80% of all cases. According to Global Burden of Disease 2021 data, approximately 55 million people worldwide are living with dementia, a figure projected to exceed 153 million by 2050 as populations age (Hu et al. 2025, Zhang et al. 2024). In the United States, an estimated 6.9 million Americans aged 65 or older are currently living with Alzheimer’s dementia, and one new case of dementia is diagnosed approximately every 3 s worldwide (2024 Alzheimer’s disease facts and figures 2024). The annual global economic cost of dementia exceeds USD 1 trillion. The earliest symptoms of AD are episodic memory deficits, followed by progressive impairment across cognitive domains. Individuals with advanced disease also develop neuropsychiatric symptoms and ultimately lose the ability to carry out basic activities of daily living.

Animal models are essential tools for preclinical AD research. Over more than three decades, an increasing number of genetically modified mouse models have been created to examine disease etiology and progression, dissect pathogenesis, and test therapeutic interventions prior to human trials. Despite this, clinical translation has been poor: many drugs that showed efficacy in animal models have failed in clinical trials. This failure has been attributed to multiple factors, including the overreliance on transgenic models carrying mutations causative for rare familial AD (fAD), which represents fewer than 5% of all cases, to test drugs intended to treat the genetically complex, polygenic late-onset AD (LOAD) affecting the remaining 95% of patients.

A significant inflection point was reached with the regulatory approvals of two anti-amyloid immunotherapies. Lecanemab received traditional FDA approval in July 2023 following the phase III CLARITY-AD trial, and European Medicines Agency (EMA) approval in late 2024. Donanemab received FDA approval in July 2024 following the TRAILBLAZER-ALZ 2 trial, and EMA marketing authorization in 2025 (Papaliagkas 2025, Dyck 2026). Both agents demonstrated statistically significant, though modest, slowing of cognitive and functional decline in biomarker-confirmed early AD, providing clinical evidence that lowering amyloid can modify disease progression. However, they also illuminate key unmet needs: the therapies are only applicable to patients in the earliest stages of the disease; they do not address (Xu et al. 2026) LOAD mechanisms beyond amyloid; and both carry a risk of amyloid immunotherapy-related adverse events (ARIA), cerebral edema and microhemorrhages, that is substantially elevated in APOEε4 carriers and individuals with concurrent cerebral amyloid angiopathy (CAA) (Dyck 2026). These limitations underscore why better mouse models of LOAD, including models of CAA and vascular contributions to cognitive impairment, are urgently needed.

The diagnostic landscape has simultaneously shifted. In 2024, the Alzheimer’s Association Workgroup published revised criteria for the biological diagnosis and staging of AD, superseding the earlier ATN framework with a comprehensive multimodal classification that defines AD by its underlying biology rather than symptoms (Jack et al. 2024). Core-1 biomarkers, amyloid PET, CSF or plasma Aβ42/40 ratio, plasma p-tau217, or p-tau181, are now considered sufficient to diagnose AD and distinguish among mixed pathologies even in cognitively unimpaired individuals. This represents a paradigm shift with direct implications for preclinical mouse models: models must now be benchmarked against biomarker-defined disease states, not only behavioral or histopathological endpoints. The availability of validated plasma biomarkers that can be measured with identical platforms in both mice and humans provides an unprecedented translational bridge.

The goal of this review is to evaluate strategies being implemented to improve the translational fidelity of mouse models for AD therapeutic development. We focus particularly on the IU/JAX/PITT MODEL-AD program (www.model-ad.org), a NIA-funded, multi-institution consortium that has systematically created, characterized, and distributed a new generation of LOAD-relevant mouse models for preclinical testing (Sukoff Rizzo et al. 2020, Oblak et al. 2026), as a case study of one systematic approach to improving model selection and validation.

## Molecular pathophysiology of AD: key targets for modeling

Two key molecules implicated in AD, tau and amyloid-β (Aβ) (Polanco et al. 2018), form fibrillar aggregates through oligomeric intermediates that are increasingly recognized as the primary neurotoxic species (Polanco et al. 2018). Tau is a microtubule-associated protein (MAPT) that, under disease conditions, accumulates in a hyperphosphorylated filamentous form in neurites as neuropil threads and in the soma as neurofibrillary tangles (NFTs) (Goedert 2015, Goedert et al. 1989). Aβ is a cleavage product of the amyloid precursor protein (APP) (Masters et al. 1985). The 42-amino acid form (Aβ42) is most prone to aggregation and forms extracellular amyloid plaques; when surrounded by dystrophic neurites rich in tau these become neuritic plaques. With advancing age, most patients also exhibit cerebral amyloid angiopathy (CAA) as perivascular amyloid deposits (Cozza et al. 2023). In addition to plaques and tangles, AD brains are characterized by profound synapse and neuronal loss, activated astrocytes and microglia, and white matter abnormalities (Duyckaerts et al. 2009).

Animal modeling of AD is complicated by the fact that a large majority of patients with amyloid and tau pathology also exhibit co-pathologies including TDP-43 inclusions, hippocampal sclerosis, Lewy body pathology, and/or vascular compromise (Young-Pearse et al. 2023). This co-pathology burden makes it exceedingly difficult to recapitulate the full human disease in any single animal model, and underscores the necessity of using multiple models to study distinct disease aspects and therapeutic targets.

Autosomal dominant (familial) forms of AD are caused by mutations in APP, PSEN1, and PSEN2, which encode proteins central to Aβ generation. However, fAD represents only 1–5% of all AD cases (Liu et al. 2025, Tanzi 2012). The majority are LOAD, arising from a complex interplay of genetic, epigenetic, and environmental factors across the lifespan. Large genome-wide association studies have now identified over 80 risk loci for LOAD (Shade et al. 2024, Shade et al. 2025, Shade et al. 2025, Verheijen and Sleegers 2018). The most significant common genetic risk factors for LOAD are the ε4 variant of apolipoprotein E (APOEε4), which confers approximately three- to four- fold increased risk in heterozygous carriers and eight- to twelvefold increased risk in homozygous carriers, and damaging variants in TREM2, which confer approximately two- to threefold increased risk (Corder et al. 1993, Guerreiro et al. 2013). Crucially, the magnitude of APOEε4 risk is not uniform across ancestral backgrounds, it is strongest in non-Hispanic white populations and substantially attenuated in African-American and Hispanic populations, a finding with important implications for model design and for ensuring diversity in preclinical studies (Griswold et al. 2021).

Genetic risk for LOAD is enriched in genes expressed in microglia and other myeloid cells, implicating innate immune responses in disease (Efthymiou and Goate 2017, Bellenguez et al. 2022). Risk genes are not restricted to microglia, however: APOE and CLU are highly expressed in astrocytes, SORL1 and APP in neurons, and BIN1 in oligodendrocytes as well as microglia, and many risk genes are expressed in endothelial cells, pericytes, and other vascular cells (Sun et al. 2023, Yang et al. 2022). Models therefore need to capture risk-gene function across several cell types. For microglial genes, species differences add a further challenge: the murine innate immune system, while broadly homologous to the human, differs in important ways including the expression and function of key AD risk genes (Mancuso et al. 2019). Capturing the human microglial risk landscape is therefore one of the central challenges driving next-generation model development (De Strooper and Karran 2026).

## How do you model AD in the mouse?

Before addressing the limitations of existing models, it is worth recognizing where mouse models have demonstrably delivered. The development of lecanemab and donanemab, the first disease-modifying therapies approved for Alzheimer’s disease, was directly enabled by decades of work in transgenic amyloid mouse models (Demattos et al. 2012, Sevigny et al. 2016, Tucker et al. 2026). These models defined the amyloid cascade hypothesis in mechanistic detail, validated anti-amyloid immunotherapy as a target class, informed antibody design and dosing strategies, and supported IND-enabling pharmacology packages that moved candidates into human trials. The vascular adverse events now recognized clinically as amyloid-related imaging abnormalities (ARIA), cerebral edema (ARIA-E) and microhemorrhages (ARIA-H), were anticipated by cerebral amyloid angiopathy and vascular lesions observed in transgenic amyloid mice treated with anti-amyloid immunotherapy, providing an early preclinical signal of a safety liability that later emerged as a central issue in clinical trials (Pfeifer et al. 2002, Racke et al. 2005). For this therapeutic strategy, mouse models were valuable.

Where the record is more sobering is beyond the amyloid axis. Despite decades of effort and hundreds of published mouse models, the clinical translation rate from mouse studies to human trials has been remarkably poor. The question of what constitutes a “good” model is therefore central to the field, and the answer depends critically on which disease mechanisms are being studied and which therapeutic target is being tested. For the purposes of this review, we define a translationally useful mouse model as one that: (1) exhibits relevant biological disease signatures (e.g., brain proteomics, blood-based biomarkers) at the correct anatomical loci; (2) expresses the target of therapeutic interest at the endogenous expression level; (3) shows cognitive or functional outcomes that can be tracked longitudinally; and (4) allows a pre-specified therapeutic window during which drug interventions can be tested at a dose and timing relevant to clinical use.

It is useful to distinguish three forms of validity that are often conflated. Construct validity refers to how faithfully a model reproduces the genetic and molecular causes of the human disease. Face validity refers to how closely the model’s phenotypes resemble features of the human disease. Predictive validity refers to whether therapeutic responses in the model anticipate responses in patients. Knock-in and genome-replacement strategies mainly improve construct validity: they remove overexpression and integration-site artifacts and introduce human risk alleles in their endogenous genomic context. Better construct validity is a reasonable prerequisite for better predictive validity, but it does not guarantee it, and whether humanized LOAD models predict clinical efficacy better than earlier models has not yet been tested prospectively. We therefore present these models as better-controlled tools for asking specific mechanistic and therapeutic questions, not as validated predictors of clinical outcome.

Rodents do not naturally develop AD plaques and NFTs. These pathological hallmarks must therefore be induced, either genetically or through exogenous delivery of pathological material. Over more than 30 years this has predominantly been achieved through transgenic overexpression of fAD-mutant genes, an approach that has yielded important mechanistic insights and supported anti-amyloid antibody development, but has a poor record of predicting clinical success for other mechanisms. As of 2025, over 280 mouse models of AD have been described in the scientific literature (Timofeeva et al. 2025), yet none fully recapitulates the complete human disease. The major conceptual and technical limitations of existing models are discussed below.

## Concerns with existing transgenic mouse models of AD

### The transgenic overexpression problem

The earliest and most widely used AD mouse models relied on random transgenic integration of human APP, PSEN1, and/or PSEN2 genes carrying fAD mutations, often driven by strong heterologous promoters such as PrP, Thy1, or CamKII. This approach generates substantially elevated expression levels of the mutant proteins, often five- to ten- fold above endogenous, which can overwhelm cellular proteostasis mechanisms independently of the mutation itself. As reviewed in detail elsewhere (Jankowsky and Zheng 2017, Onos et al. 2016, Sasaguri et al. 2017), several studies have demonstrated that the insertion of large transgenic constructs frequently disrupts endogenous gene function at the integration site, creating additional confounds that are difficult to distinguish from the intended transgene effect. The hAPP-J20 line has been shown to carry an insertion into the Zbtb20 gene, likely contributing to the seizure phenotype seen in that model (Gulbranson et al. 2021). These findings indicate that phenotypic characterization of transgenic lines must account for integration-site effects, and that knock-in approaches that avoid these confounds are preferable where the question does not require overexpression.

A second fundamental problem is conceptual: virtually all widely used AD mouse models express mutations causative for fAD, a genetically distinct, rare syndrome affecting 1–5% of patients. Drugs identified as beneficial in fAD models may be effective specifically because they target the fAD-specific mechanism being modeled (e.g., reducing overproduced Aβ from an APP transgene) rather than the more complex and diverse mechanisms underlying LOAD. This may help explain why some anti-amyloid approaches that were effective in transgenic models failed in clinical trials: overexpression models report efficacy against a heavy, fAD-driven amyloid burden and may say little about the mechanisms that drive LOAD. Conversely, drugs that target LOAD-specific mechanisms, such as APOE lipidation, TREM2 signaling, or microglial activation pathways, cannot be properly evaluated in a model that lacks these genetic contexts. As discussed below, aging is the strongest risk factor for AD, but is not incorporated in Tg models.

### Limitations of specific transgenic lines

The 5XFAD mouse is among the most widely used models due to its aggressive and early amyloid phenotype, plaques beginning at 2 months of age, and is therefore popular for drug screening. However, comprehensive evaluation by MODEL-AD demonstrated that 5XFAD primarily induces an immune/inflammatory transcriptional response and does not recapitulate the broader molecular heterogeneity seen in human LOAD (Oblak et al. 2021). Prophylactic evaluation of the BACE1 inhibitor verubecestat in 5XFAD mice showed target engagement and amyloid reduction but did not reduce cognitive deficits (Oblak et al. 2022), illustrating that amyloid reduction does not automatically translate to functional benefit even in this model. The 3xTg-AD and APP/PS1 models, while incorporating both amyloid and tau components, similarly rely on overexpression of fAD mutations and lack LOAD genetic risk context.

## Approaches to improved modeling in mice

### Knock-in and CRISPR-based engineering

With knock-in and CRISPR-based approaches, human coding sequences and/or genetic risk variants can be introduced into the endogenous mouse locus, leveraging the endogenous promoter and regulatory regions to drive physiologically appropriate expression throughout the lifespan. This is the central engineering principle underpinning all MODEL-AD models and represents a substantial conceptual advance over transgenic approaches. Because expression is driven by the endogenous locus, knock-in models do not produce supraphysiological protein levels and do not carry integration-site mutagenesis risks.

The APP^NL−G−F^ (NL-G-F) knock-in model, incorporating three fAD mutations (Swedish, Arctic, Iberian) along with a humanized Aβ region, demonstrated that physiological-level expression of human amyloid sequences is sufficient to drive robust plaque pathology without transgene overexpression (Saito et al. 2014). More recently, the APP^SAA^ knock-in model, created with three fAD mutations (Swedish, Arctic, Austrian), was developed specifically for pharmaceutical preclinical testing. This model, available without restrictions on use from The Jackson Laboratory as JAX #034711, exhibits extensive amyloid pathology, elevated phosphorylated tau, a disease-associated microglia (DAM) transcriptomic signature, microglial metabolic disruption, and structural brain changes detectable by MRI (Xia et al. 2022, Sloan et al. 2026). The APP^SAA^ model is being comprehensively characterized by MODEL-AD as a standard platform for testing anti-amyloid and neuroinflammation-targeted therapeutics. However, these models mimic fAD, not LOAD.

For tauopathy modeling, differential splicing of human MAPT (producing six isoforms with a 1:1 ratio of 3R:4R tau) versus mouse Mapt (producing almost exclusively 4R-tau isoforms in adult brain) makes human MAPT biology fundamentally inaccessible in standard mouse models. Genome replacement (GR) approaches, in which large genomic regions including flanking regulatory sequences are substituted with their human counterpart, are essential for recapitulating the correct isoform biology. By replacing the entire mouse *Mapt* locus with the human *MAPT* H1 haplotype (the major LOAD/tauopathy-associated haplotype), mice expressing the correct 3R:4R isoform ratio were created (Benzow et al. 2024). These GR-MAPT mice provide a foundation onto which LOAD risk variants, or combinations with amyloid models, can be introduced (Benzow et al. 2024, Morito and Watamura 2025). Models with mutations associated with discrete tauopathies (e.g. FTD, Pick’s disease) have been inserted into the MAPT-GR locus to drive discrete tau pathologies and are available through the Jackson Laboratory, again without restrictions on use.

Knock-in strategies also enable study of non-coding regulatory variants, which constitute a substantial fraction of LOAD genetic risk. Variants residing in promoters, enhancers, or splicing regulatory elements cannot be studied in transgenic overexpression models at all. Comparing mice with and without specific non-coding variants at the APOE locus, for example, allows attribution of ancestry-specific risk differences to specific regulatory sequences rather than to protein-coding changes.

## Late-onset AD vs. familial AD: the central distinction

The distinction between fAD and LOAD is not merely academic: it defines the scope of therapeutic relevance of any preclinical model. fAD is caused by dominant mutations in APP, PSEN1, and PSEN2 that dramatically increase Aβ42 production. LOAD is caused by a combination of common genetic risk variants (most importantly APOEε4 and TREM2 variants), rare coding variants across the AD risk loci identified by GWAS, epigenetic modifications, environmental exposures, and the biology of aging itself.

MODEL-AD’s strategy has been to build LOAD mouse models from the genetic ground up, beginning with the two most significant common LOAD risk genes. The LOAD1 model (JAX #028709: B6.APOEε4.Trem2*R47H) was the first in the series, incorporating double-homozygous knock-in alleles of humanized APOE4 and the R47H missense variant in mouse *Trem2*. Comprehensive cross-sectional and longitudinal characterization of LOAD1 mice revealed that, while these two risk alleles were insufficient on their own to drive overt amyloid or tau pathology at 24 months, they produced a biologically meaningful “sensitized background” characterized by altered neuroinflammation, differential splicing of neuronal genes that mimics patterns seen in human AD, changes in cerebrovascular blood flow and glucose uptake, and a transcriptional profile that correlates with human AMP-AD co-expression modules (Oblak et al. 2020). This provided the empirical justification for using LOAD1 as a platform onto which additional risk alleles could be added.

The LOAD2 model (JAX #030670: B6.APOE4.Trem2*R47H.hAβ) added humanized amyloid-β sequences to the LOAD1 background, creating a triple-homozygous strain. The humanized Aβ knock-in allele (hAβ; also known as App^hAβ^) converts the three amino acid differences between mouse and human Aβ to the human sequence, making the peptide more amyloidogenic. Comprehensive characterization of LOAD2 across multi-site cohorts, aged at 4, 12, 18, and 24 months on either control diet (CD) or high-fat/high-sugar diet (HFD), yielded one of the most extensive characterizations of a LOAD-based mouse model to date (Kotredes et al. 2024). Results showed that age was the primary driver of gene expression changes, while HFD exacerbated AD-relevant molecular changes including lipid metabolism, oxidative stress, and apoptotic pathway dysregulation. By 18 months, LOAD2 + HFD mice exhibited significant cortical neuron loss, elevated insoluble brain Aβ42, increased plasma NfL (a clinically validated neurodegeneration biomarker), age-dependent brain region volume reduction and neurovascular uncoupling by in vivo imaging, and deficits in touchscreen-based cognitive tasks (Kotredes et al. 2024). This neurodegeneration occurred independently of frank amyloid plaque formation at the ages studied, representing a pathological state analogous to the preclinical or early prodromal phase of LOAD. This makes LOAD2 + HFD a useful model for testing therapeutics that target inflammation, lipid metabolism, neurodegeneration, and vascular function in the prodromal stage of disease.

Most recently, the LOAD3 model has been introduced, adding humanized MAPT (hMAPT, H1 haplotype genome replacement) to the LOAD2 background to create a triple-humanized platform (APOE4.hAβ.hMAPT; JAX #037778). LOAD3 and its APOE3 counterpart (APOE3.hAβ.hMAPT, created in collaboration with the BU/JAX/IU TOX-AD center) are being comprehensively characterized under environmental stressors (e.g. high-fat diet, heavy metals) with the goal of capturing tau isoform biology inaccessible in current models. The availability of both APOE3 and APOE4 versions allows direct comparison of the most consequential common AD risk allele in a full LOAD genetic context.

The fAD–LOAD distinction is not a ranking of models. fAD-based models, including transgenic lines, remain the most appropriate tools for many questions. They develop robust parenchymal and vascular amyloid within a practical timeframe, they supported the development of the approved anti-amyloid antibodies, and they remain the most tractable systems for studying amyloid clearance, CAA, and ARIA-like vascular responses to immunotherapy (Pfeifer et al. 2002, Racke et al. 2005). Amyloid knock-in models such as APP^SAA^ and APP^NL−G−F^ provide robust amyloid biology without overexpression artifacts. LOAD platform models are better suited to mechanisms that fAD models lack, such as APOE-dependent lipid and vascular biology, microglial risk-gene signaling, and gene x environment interactions, and to the early, pre-plaque phase of disease. Crossing LOAD risk alleles onto amyloid knock-in backgrounds can combine these strengths. Model choice should therefore follow from the therapeutic question; Table 1 summarizes the intended uses and limitations of commonly used models.

**Table 1:** Comparison of MODEL-AD LOAD platform models with commonly used amyloid models (genotype, reported phenotypes, intended use, and limitations)

Model	Genotype (JAX stock)	Key phenotypes reported	Best suited for	Main limitations
LOAD1	APOE4 KI; Trem2*R47H; homozygous (#028709)	No plaques or tangles to 24 months; altered neuroinflammation; neuronal splicing changes resembling human AD; altered cerebral blood flow and glucose uptake; correlation with AMP-AD modules	Sensitized background for adding risk alleles; APOE4- and TREM2-dependent mechanisms; early, pre-pathology biology	Trem2*R47H reduces Trem2 expression in mouse (not in human); modest phenotypes; no amyloid or tau endpoints
LOAD2 (± HFD)	LOAD1 + humanized Aβ (App hAβ) (#030670)	Age-driven transcriptomic change; with HFD by 18 months: cortical neuron loss, raised insoluble Aβ42 and plasma NfL, volume loss, neurovascular uncoupling, touchscreen deficits; no frank plaques at ages studied	Prodromal, non-amyloid mechanisms (inflammation, lipid metabolism, vascular function, neurodegeneration); gene x environment studies	Long aging periods, small effect sizes, large cohorts; HFD effects depend on diet, sex, housing; cannot test plaque-directed therapies; murine tau
LOAD3	APOE4 KI; hAβ; hMAPT (H1) genome replacement (#037778); APOE3 counterpart available (#40313)	Characterization ongoing (± HFD, heavy metals)	Human tau isoform biology in a LOAD context; APOE3 vs. APOE4 comparisons; tau-directed therapies once phenotypes are defined	Phenotypes not yet published; timing of any tau pathology unknown
APP^SAA^	App KI with Swedish, Arctic, Austrian mutations (#034711)	Robust plaques, raised p-tau, DAM signature, microglial metabolic changes	Anti-amyloid and neuroinflammation PK/PD and efficacy without overexpression artifacts	fAD mutations; lacks LOAD risk context unless crossed with APOE4
5XFAD	APP/PS1 transgenic carrying 5 fAD mutations	Plaques from 2 months; strong inflammatory response; neuron loss	Rapid amyloid target engagement; amyloid–microglia interactions	Overexpression; limited molecular overlap with LOAD; amyloid lowering without cognitive benefit (verubecestat)

## Incorporating additional LOAD risk variants

Beyond the LOAD1/2/3 series, MODEL-AD has systematically created and validated mouse lines carrying a broad panel of individual LOAD risk variants, using LOAD1 or LOAD2 as sensitized platform backgrounds. We have recently reported in vivo validation of 11 candidate LOAD risk variants knocked into conserved mouse loci, assayed by transcriptional analysis at 4 and 12 months. Risk variants in Abca7, Mthfr, Plcg2, and Sorl1 loci modeled molecular signatures characteristic of clinical AD, including alterations in cell-cycle, protein-folding, fatty acid metabolism, and synaptic vesicle gene expression respectively. Notably, these molecular signatures are distinct from those of traditional transgenic models such as 5XFAD, which predominantly drive inflammatory gene expression responses to amyloid.

Additional models incorporating non-coding regulatory variants at conserved loci have been created at Adamts4, Bin1, Cd2ap, Epha1, Ptk2b, and Scimp, providing tools for studying the substantial fraction of LOAD risk residing in non-coding regulatory regions (Sasner et al. 2024). A newly characterized series of models expressing variants at the IL1RAP locus demonstrates LOAD-relevant endophenotypes including transcriptional alterations in immune signaling pathways, further expanding the toolkit (Kotredes et al. 2024). Two Plcg2 variants have been characterized in the LOAD2 background with and without HFD, providing both a risk model (Plcg2*M28L) and a protective variant (Plcg2*P522R) that can be used to bound therapeutic target windows.

Across all of these models, the MODEL-AD consortium has emphasized open science: all mouse strains are available from The Jackson Laboratory, all phenotypic data are deposited in the AD Knowledge Portal (adknowledgeportal.org), and all protocols are publicly accessible. The MODEL-AD Explorer tool, hosted on the AD Knowledge Portal, provides interactive access to transcriptomic, proteomic, metabolomic, and imaging data across all characterized models, enabling researchers to select the most appropriate model for a given therapeutic target before committing to in vivo studies.

### Limitations of next-generation LOAD models

Knock-in LOAD models avoid several artifacts of transgenic overexpression, but they have their own limitations, which should guide how they are used.

First, their phenotypes are modest and develop slowly. LOAD1 mice do not develop amyloid plaques or neurofibrillary tangles by 24 months, and LOAD2 mice develop neurodegenerative changes without frank plaque formation at the ages studied. The humanized Aβ allele alone produces only limited, late amyloid pathology (Baglietto-Vargas et al. 2021). These models therefore cannot test therapies that act on established amyloid or tau aggregates, and efficacy studies in them need long aging periods and large cohorts to detect small effects, which makes them costly.

Second, a humanized allele does not necessarily behave like the human variant. The Trem2*R47H allele in LOAD1 and LOAD2 causes aberrant splicing and reduced Trem2 mRNA and protein in mice, which is not seen in human carriers (Xiang et al. 2018). Part of the phenotype may therefore reflect reduced Trem2 dosage rather than the human missense mechanism. Each new risk allele needs similar validation at the transcript, protein, and functional levels.

Third, the cellular and physiological context is still murine. Humanized APOE, APP, and MAPT are expressed in mouse cells, interact with mouse partner proteins, and operate within mouse immune and vascular systems over a lifespan of two to three years. Many GWAS loci lack a clear mouse ortholog or conserved regulatory sequence, and polygenic risk, the combined effect of many small-effect variants, cannot yet be reproduced by adding one or a few alleles at a time.

Fourth, most characterization has been done on the C57BL/6J background, with the limitations discussed below, and the effects of environmental modifiers such as high-fat diet depend on diet composition, duration, sex, and housing, which makes results hard to compare across laboratories.

Finally, and most importantly for preclinical testing, no LOAD platform model has yet been benchmarked prospectively against clinical trial outcomes. Their value for predicting therapeutic efficacy is a hypothesis that still has to be tested.

## Gene x environment interactions: incorporating environmental risk

Aging itself is the dominant risk factor for LOAD, but aging does not occur in a vacuum: decades of dietary habits, physical activity patterns, vascular risk factor burden, and environmental exposures all modulate the trajectory from normal aging to cognitive impairment. The MODEL-AD Consortium has been a leader in incorporating environmental risk factors into LOAD mouse models to better reflect the multifactorial nature of human LOAD.

The most extensively characterized gene x environment manipulation is the combination of LOAD2 genetics with chronic high-fat/high-sugar diet (HFD), modeling the metabolic syndrome risk that is epidemiologically associated with LOAD. As described above, LOAD2 + HFD produces age-related neurodegeneration independent of frank amyloid pathology, with plasma NfL, cortical neuron loss, and imaging biomarkers that parallel clinical measurements (Kotredes et al. 2024). The LOAD2.Plcg2*M28L model has also been evaluated with HFD, demonstrating that the protective Plcg2 variant attenuates diet-induced neurometabolic and vascular dysfunction.

These results should be interpreted with the limits of the dietary model in mind. High-fat/high-sugar feeding in mice reproduces some features of metabolic syndrome, such as obesity, insulin resistance, and dyslipidemia, but not the decades-long exposure, heterogeneity, or comorbidities of human metabolic disease. Diet effects depend on composition, age at start, duration, and sex, and peripheral metabolic changes can confound central endpoints. HFD models are therefore best seen as a controlled metabolic stressor for testing gene x environment interactions, rather than as a model of human metabolic syndrome.

Extending beyond diet, the MODEL-AD exposome study has examined the effects of toxic metal exposures on LOAD model mice. Chronic inorganic arsenic exposure, an environmental stressor associated with increased AD risk in epidemiological studies, has been evaluated in LOAD2.Mthfr*677C > T mice, a model that incorporates a LOAD risk variant in methylenetetrahydrofolate reductase (*Mthfr*) which increases homocysteine levels and disrupts methylation pathways. This gene x environment combination captures a biologically plausible interaction between a one-carbon metabolism genetic risk variant and an environmental exposure that perturbs the same pathway.

### Modeling vascular contributions to AD

Vascular contributions to cognitive impairment and dementia (VCID) represent another major environmental x genetic interaction domain. Hypertension, cerebrovascular disease, and diabetes mellitus all substantially increase LOAD risk and are present in many AD patients as comorbidities. The wild-derived WSB/EiJ background has been identified as exhibiting elevated CAA in the presence of amyloid-generating alleles (Onos et al. 2019), and current work from the MODEL-AD Center is exploiting WSB and other wild-derived backgrounds to model vascular AD phenotypes including CAA, directly relevant to the ARIA risk associated with anti-amyloid immunotherapy (Yang et al. 2021).

Vascular dysfunction is among the earliest and most common changes in LOAD, and it is central to the safety of anti-amyloid therapy. Breakdown of the blood–brain barrier (BBB) in the hippocampus is detectable early in cognitive impairment and in APOE4 carriers, independent of amyloid and tau (Montagne et al. 2020). Most people with AD also have small vessel disease, including arteriolosclerosis, microinfarcts, and white matter hyperintensities, as well as reduced cerebral blood flow and cerebrovascular reactivity (Sweeney et al. 2019). Human vascular single-nucleus atlases show that many LOAD risk genes are expressed in endothelial cells, pericytes, smooth muscle cells, and perivascular fibroblasts (Sun et al. 2023, Yang et al. 2022).

Mouse models capture parts of this biology. APOE4 targeted-replacement mice show pericyte loss and BBB leakage through a cyclophilin A–MMP9 pathway (Bell et al. 2012), and LOAD1 and LOAD2 mice show altered cerebral blood flow and neurovascular uncoupling. CAA-prone models, including Tg-SwDI mice (Davis et al. 2004) and amyloid alleles on the WSB/EiJ background (Marola et al. 2026), allow vascular amyloid to be studied. Hypertension and diabetes models, and chronic cerebral hypoperfusion by bilateral carotid artery stenosis (Shibata et al. 2004), can be combined with AD risk alleles to study mixed vascular and AD pathology.

These approaches have limits. Mice have relatively little white matter, rarely develop spontaneous small vessel disease, and differ from humans in cerebrovascular anatomy. Endpoints that can be measured in both species include dynamic contrast-enhanced MRI for BBB permeability, arterial spin labeling for cerebral blood flow, cerebrovascular reactivity to hypercapnia, diffusion MRI of white matter, and susceptibility-weighted imaging for microhemorrhages. One important gap for current therapeutics is a validated model of ARIA risk as a function of APOE genotype and CAA burden; combining humanized APOE4 with CAA-prone amyloid alleles or genetic backgrounds is one route to such a model.

### Diverse genetic backgrounds

Most published AD mouse models have been generated on, or made congenic on, the C57BL/6J (B6J) background. While B6J is the standard laboratory background due to its well-characterized genome and wide availability of genetic resources, it represents a single point in the enormous genetic space of the mouse population, and human population. The choice of background has been shown to substantially influence AD-relevant phenotypes.

Systematic studies by Onos and colleagues demonstrated that wild-derived genetic backgrounds dramatically alter Aβ deposition patterns, microglial morphology and reactivity, and the severity of cognitive deficits in amyloid-generating models (Onos et al. 2019). The CAST/EiJ strain, for example, exhibited dramatically reduced plaque burden compared to B6J even when carrying the same amyloid-generating alleles. The WSB/EiJ background promotes CAA rather than parenchymal plaques. Natural genetic variation was also shown to be a powerful determinant of microglial heterogeneity: the number, morphology, and transcriptional state of microglia varied across backgrounds independently of any AD allele (Yang et al. 2021), implying that single-background studies may miss the range of microglial responses that underlie human interindividual variability in AD progression and treatment response.

The Collaborative Cross (CC) and Diversity Outbred (DO) populations offer systematic approaches to capturing genetic diversity in mouse preclinical studies. CC mice are a panel of inbred strains derived from eight diverse founder strains, capturing approximately 90% of common mouse genetic variation. DO mice are outbred, genetically unique individuals derived from the same eight founders, providing a model system that better parallels the genetic heterogeneity of human populations. Introducing LOAD knock-in alleles into CC or DO backgrounds, as MODEL-AD and others are pursuing, would allow detection of genetic modifiers, enable pharmacogenomic studies, and substantially increase the face validity of models for preclinical therapeutic testing.

## The AD knowledge portal and open science infrastructure

A crucial but sometimes underappreciated aspect of MODEL-AD’s contribution to the field is its open science infrastructure. All model characterization data, analysis workflows, protocols, and metadata are deposited in the AD Knowledge Portal (adknowledgeportal.synapse.org), a centralized repository established by Sage Bionetworks and funded by the NIA to make both clinical and animal model data sets available. The portal currently hosts multi-omics data (transcriptomics, proteomics, metabolomics, lipidomics), imaging data, neuropathology, biomarker measurements, and behavioral outcomes from over 30 mouse models, all processed using harmonized analytical pipelines that allow direct cross-model and cross-study comparison.

The MODEL-AD Explorer tool, accessible through the portal, provides an interactive interface for browsing and downloading model characterization data, including RNA-seq data processed through a common workflow to generate harmonized differential expression results across all models. This enables a researcher designing a preclinical study to query which existing models show transcriptional signatures most concordant with the target biology of their drug candidate, or to identify which models express a given target gene at levels likely to support pharmacological modulation. All mouse strains are concurrently made available through The Jackson Laboratory, ensuring global accessibility.

This open-science model stands in stark contrast to the historical siloing of academic mouse model data, which contributed to irreproducibility and the failure to systematically compare models across the field.

## Inducible protein aggregation through non-genetic methods

In addition to germline knock-in models, inducible models using stereotaxic injection have continued to provide mechanistic insight into seeded aggregation and spreading, which is mechanistically relevant to the prion-like propagation of tau and Aβ pathology. Injection of synthetic Aβ aggregates into tau transgenic or humanized tau mice accelerates NFT formation, providing support for the amyloid cascade hypothesis and an experimental tool to study the downstream consequences of focal Aβ seeding. Stereotaxic injection of brain lysates from AD patients into knock-in mice has revealed a highly organized multistep process by which Aβ plaques facilitate rapid amplification of tau seeds into large aggregates (Clavaguera et al. 2009, He et al. 2018). AAV-mediated brain delivery of AD-relevant transgenes, including mutant tau, APP fragments, or gain- and loss-of-function variants of microglial genes such as Trem2 and Plcg2, provides a rapid alternative to germline modification for testing hypotheses in vivo. Advantages of injection-based approaches include speed, localizability of expression or pathology to defined brain circuits, and independence from transgene background effects. Disadvantages include the requirement for invasive neurosurgery, batch-to-batch variability in injection material, non-uniform distribution of expression or pathology, and the inability to generate stable heritable lines for large-scale preclinical testing.

## Chimeric and xenotransplantation models

A complementary strategy to fully humanized knock-in lines is the generation of chimeric mice carrying human cell populations within an otherwise murine brain. These models address a key limitation of knock-in approaches: even with humanized risk alleles, resident CNS cell types remain murine in origin, and species differences in transcriptional and metabolic programs can limit translational fidelity. Xenotransplantation approaches engraft human iPSC-derived microglial progenitors or hematopoietic stem cells into the neonatal or adult brain of immunodeficient mouse strains (e.g., *Rag2⁻/⁻Il2rg⁻/⁻*, MITRG/MISTRG backgrounds, often combined with humanized *CSF1* knock-in to support myeloid engraftment and survival). Engrafted human microglia adopt ramified morphology, occupy parenchymal niches, and respond to amyloid and tau pathology with transcriptional signatures more closely resembling human disease-associated microglial states than their murine counterparts (Mancuso et al. 2019, Coburn et al. 2025, Hasselmann et al. 2019, Mancuso et al. 2024). This makes chimeric models particularly well suited to studying human-specific genetic risk variants in a more authentic cellular context, and to dissecting cell-autonomous versus environmental (host) contributions to microglial phenotype. Limitations should be noted: engraftment efficiency and chimerism level vary by line and protocol, host strains typically lack adaptive immunity (precluding study of neuroimmune crosstalk involving T/B cells), and the surrounding neuronal/astrocytic/vascular compartment remains murine, so non-cell-autonomous signaling is still cross-species. Engraftment timing also constrains experimental windows relative to fully knock-in models, which can be aged and crossed conventionally. Overall, chimeric/xenotransplantation models occupy a useful middle ground between humanized knock-in mice and human iPSC organoid/MPS systems, offering in vivo context for human cells without the scalability of in vitro platforms, thus they are complementary rather than a replacement for the knock-in strategies discussed elsewhere in this review.

## Translational value of mouse models: aligning preclinical and clinical endpoints

### Blood-based biomarkers as cross-species translational endpoints

One of the most significant advances enabling translational mouse studies has been the validation of blood-based biomarkers for AD that reflect underlying neuropathology with high sensitivity and specificity, and that can, for some analytes, be measured with analogous assay platforms in mice and humans. The 2024 NIA-AA revised criteria formally recognize plasma p-tau217 as a Core-1 biomarker sufficient to diagnose AD biologically, even in cognitively unimpaired individuals (Jack et al. 2024). In a landmark multicenter validation study, plasma p-tau217 demonstrated the strongest ability to discriminate between Aβ/tau stages; plasma GFAP, an early marker of astrocyte reactivity, has been associated with the relationship between Aβ and subsequent tau accumulation; and plasma NfL is most closely associated with neurodegeneration and predicts cognitive decline. These biomarkers have been measured in the LOAD2 and other MODEL-AD mouse models at 4, 12, 18, and 24 months of age, providing stage-specific reference ranges and allowing comparison with biomarker trajectories in preclinical human studies. Plasma p-tau217 has also been formally proposed as a primary endpoint for trials in preclinical AD (Ferreira et al. 2026), which makes it an attractive endpoint for aligning preclinical and clinical studies in models that express human tau.

Cross-species equivalence nonetheless depends on the analyte. NfL is highly conserved, and the same assay platforms can be used in both species. GFAP is also well conserved. Plasma p-tau217 is more complicated: mouse and human tau differ in sequence, so measurements in models with murine tau require species-specific assays whose relationship to the human marker has to be established. Mouse plasma volumes, reference ranges, and the relationship between plasma levels and brain pathology also differ from those in humans. These markers are therefore best used as aligned endpoints that must be calibrated within each model, rather than as interchangeable measures.

Multi-omics profiling, encompassing transcriptomics (bulk RNA-seq, single-cell RNA-seq), proteomics, metabolomics, and lipidomics, provides a further dimension of translational comparability. A central deliverable of the MODEL-AD program is the generation of multi-omics data from all mouse models aged to multiple timepoints, processed using the same bioinformatic workflows used to analyze human AMP-AD data. This enables direct module-level comparison of mouse model transcriptional states with human AD transcriptional states, identifying which models, and at which ages, most closely resemble specific phases of human disease.

### In vivo imaging as a translational phenotyping tool

Structural MRI, amyloid PET, tau PET, and FDG-PET are now core outcome measures in human AD clinical trials. The MODEL-AD phenotyping pipeline incorporates all four modalities in aged mouse cohorts, with imaging data generated at Indiana University’s Stark Neurosciences Research Institute using both small-animal scanners and human-equivalent radioligands where available. Tau PET is of limited use in models with murine tau, which do not form tangles, and is most relevant to models expressing human MAPT. Structural MRI provides brain volume measurements and hippocampal atrophy indices that closely parallel the volumetric biomarkers used in clinical trials such as CLARITY-AD. FDG-PET provides a proxy for synaptic and neuronal metabolic activity. Amyloid PET, while technically challenging in mice due to the small brain volumes and lower plaque densities relative to humans, is being optimized for use in APP^SAA^ mice.

Neurovascular coupling, the relationship between neural activity and local blood flow, measured in mice using fMRI or optical imaging, has been found to be altered in LOAD2 + HFD mice in an age-dependent manner (Kotredes et al. 2024), mirroring the neurovascular dysfunction seen in early human LOAD and VCID. This provides a potentially powerful translational endpoint for therapeutics targeting the neuroinflammatory or vascular components of LOAD.

### Cognitive and behavioral assessment

Cognitive testing in mice has historically relied on paradigms such as the Morris water maze, fear conditioning, and novel object recognition. While these tasks reveal learning and memory deficits under certain conditions, they have significant limitations: they are highly confounded by non-cognitive factors such as motor performance, anxiety, visual acuity, and motivation; they use stimuli (cold water, electric shock, novel odors) that are not ethologically relevant to the rodent sensory world; and they have no equivalent in human clinical assessment. The predictive validity of these assays for detecting clinically translatable drug effects has been repeatedly questioned.

Operant touchscreen-based cognitive testing platforms, originally developed by Bussey and Saksida, offer a more translatable alternative. The touchscreen platform uses visual stimuli presented on a screen to assess cognitive domains including pattern separation, paired associate learning, sustained attention, and reversal learning, using task structures analogous to the Cambridge Neuropsychological Test Automated Battery (CANTAB) used in human clinical research. MODEL-AD uses touchscreen testing as a standard component of its deep phenotyping pipeline, and LOAD2 + HFD mice showed significant deficits in touchscreen task acquisition at 18 months of age, indicating its sensitivity in this LOAD model. Because the same testing logic can be applied across species, touchscreen data from mice can be interpreted alongside CANTAB data from human cohorts, although direct quantitative comparison across species has not been established.

Touchscreen tasks improve the alignment of task design across species, but evidence that drug effects on mouse touchscreen performance predict effects in patients is still limited. Performance also depends on food restriction, motivation, and training history. This matters for diet studies: the touchscreen acquisition deficits in LOAD2 + HFD mice could partly reflect altered food-reward motivation or metabolic state, and should be interpreted alongside control measures of motivation and motor performance.

### Evidence for predictive validity

The most direct test of a preclinical model is whether it anticipated clinical outcomes. For anti-amyloid antibodies, the record of transgenic amyloid models is mixed but informative. Murine versions or precursors of the approved antibodies reduced amyloid in transgenic mice: mE8, the murine precursor of donanemab, in PDAPP mice (Demattos et al. 2012); mAb158, the murine version of lecanemab, in tg-ArcSwe mice; and aducanumab in Tg2576 mice (Sevigny et al. 2016). These amyloid-lowering effects were reproduced in patients, and microhemorrhages in immunized APP23 and PDAPP mice anticipated ARIA-H (Pfeifer et al. 2002, Racke et al. 2005).

The same models, however, overpredicted cognitive benefit for many compounds. They did not anticipate the cognitive worsening seen in patients with the γ-secretase inhibitor semagacestat (Doody et al. 2013) or with BACE inhibitors such as verubecestat (Egan et al. 2018); in 5XFAD mice, verubecestat lowered amyloid without improving cognition. One interpretation is that transgenic amyloid models are better suited to modeling prevention than treatment of established disease (Zahs and Ashe 2010).

Comparable data do not yet exist for knock-in LOAD models. They are recent, and few compounds have been tested in them and then in patients. Establishing predictive validity will require testing clinically characterized drugs, both successes and failures, in these models under trial-like conditions, and reporting whether the models tell them apart. Until such data exist, the case for LOAD platform models in preclinical testing rests on construct validity and on alignment of biomarker endpoints with the clinic, not on demonstrated predictive validity.

## Principles of rigorous preclinical study design

For mouse model data to have maximum predictive value, preclinical studies must be designed with the same rigor expected of clinical investigations. The following principles, now advocated by MODEL-AD and others as the standard for preclinical AD studies, reflect lessons learned from decades of poor translational outcomes:

Match the model to the mechanism: confirm that the mouse model expresses the biological target of therapeutic interest at physiologically relevant levels and in the relevant cell types before committing to efficacy testing.

- Treat at the right age: ensure that the therapeutic window is defined by confirmed presence of the target pathology at an appropriate disease stage. Treating young, pre-symptomatic mice with therapeutic intended for patients with established pathology is not translational.
- Confirm target engagement in vivo: establish pharmacokinetic–pharmacodynamic (PK/PD) relationships with quantitative measurements of target occupancy or downstream pathway modulation in the relevant tissue, in the model and age being used.
- Power for sex differences: include both sexes with adequate power to detect sex x treatment interactions, which are frequently observed in LOAD models given the sex-differential effects of APOE4.
- Randomize and blind: use computer-generated randomization and treatment-blinded outcome assessment as standard. These are mandatory for all MODEL-AD preclinical studies.
- Pre-specify endpoints and analyses: register the primary endpoint, sample size calculation, and statistical analysis plan before the study is unblinded.
- Use clinically aligned outcomes: prioritize plasma biomarkers (NfL, p-tau, GFAP), imaging (MRI volumes, PET), and touchscreen cognition over rodent-specific assays with poor translational track records.
- Follow ARRIVE 2.0 guidelines: report all elements specified in the ARRIVE 2.0 guidelines for animal research, including health monitoring, inclusion/exclusion criteria, and data availability.

Many of these elements have historically been missing from published AD preclinical studies, contributing to the irreproducibility crisis and the high clinical failure rate. MODEL-AD has implemented all these principles as required components of its phenotyping and efficacy testing programs, and advocates for their adoption across the field (Sukoff Rizzo et al. 2020, Percie du Sert et al. 2020).

## Conclusions

The past five years have witnessed a genuine inflection point in Alzheimer’s disease research. The first approvals of disease-modifying therapies, lecanemab and donanemab, have provided clinical evidence that lowering amyloid can modify disease progression, while simultaneously demonstrating the modest effect sizes achievable by targeting amyloid alone in patients with established disease. This dual message makes the case compellingly for two parallel priorities: earlier intervention, and targets beyond amyloid. Both priorities demand better mouse models.

The MODEL-AD program and related efforts have expanded the tools available to the field. The LOAD1, LOAD2, and LOAD3 platform mice, together with a growing library of individual risk-variant models, introduce major LOAD risk alleles at physiological expression levels. Systematic, multi-site, multi-omics characterization across the lifespan, with all data openly available through the AD Knowledge Portal, gives investigators an evidence base for choosing a model that was not previously available. The finding that LOAD2 + HFD mice develop neuronal loss, plasma NfL elevation, brain volume loss, and cognitive deficits without amyloid plaques provides a platform for testing therapies aimed at non-amyloid mechanisms. These models have limitations, and their ability to predict clinical efficacy has not yet been established.

The 2024 NIA-AA revised diagnostic framework, with its recognition of plasma p-tau217 as a Core-1 diagnostic biomarker and its biological staging of AD across the full disease trajectory, provides the conceptual scaffolding that mouse models must now match. Blood-based biomarkers such as NfL and GFAP, and p-tau217 in models expressing human tau, can now be measured with analogous platforms in mice and humans, allowing biomarker-level comparisons across species that must still be calibrated within each model.

The main advance, therefore, is not a single faithful mouse model of LOAD, but rather, due to the disease heterogeneity, the delivery of a panel of well characterized models to the field. It is a set of better-controlled tools and a framework for selecting and validating models for specific human mechanisms and therapeutic questions. fAD-based models remain the right choice for amyloid, CAA, and ARIA biology, and LOAD platform models for APOE-, microglial-, vascular-, and environment-dependent mechanisms in early disease. In every case, the choice should be matched to the target, supported by in vivo target engagement, and tested with clinically aligned endpoints under trial-like rigor. The next step is to test whether this approach improves prediction: evaluating drugs with known clinical outcomes in these models, and reporting whether the models tell successes from failures that mirror clinical trial standards. The tools now exist to execute this shift. The field’s willingness to adopt them will determine whether the next generation of AD therapeutic candidates will finally translate from mouse to medicine. 

## Data Availability

No datasets were generated or analysed during the current study.

## Declarations

### Statements and Declarations

MS, CRB, AO are supported by the MODEL-AD grant through the National Institute on Aging (AG054345).
